# Examining Care Planning Efficiency and Clinical Decision Support Adoption in a System Tailoring to Nurses’ Graph Literacy: National, Web-Based Randomized Controlled Trial

**DOI:** 10.2196/45043

**Published:** 2023-08-11

**Authors:** Yingwei Yao, Karen Dunn Lopez, Ragnhildur I Bjarnadottir, Tamara G R Macieira, Fabiana Cristina Dos Santos, Olatunde O Madandola, Hwayoung Cho, Karen J B Priola, Jessica Wolf, Diana J Wilkie, Gail Keenan

**Affiliations:** 1 University of Florida College of Nursing Gainesville, FL United States; 2 University of Iowa College of Nursing Iowa City, IA United States

**Keywords:** clinical decision support, nurse decision-making, nurse care planning, simulation, remote testing, tailored interfaces, graph literacy, cognitive workload

## Abstract

**Background:**

The proliferation of health care data in electronic health records (EHRs) is fueling the need for clinical decision support (CDS) that ensures accuracy and reduces cognitive processing and documentation burden. The CDS format can play a key role in achieving the desired outcomes. Building on our laboratory-based pilot study with 60 registered nurses (RNs) from 1 Midwest US metropolitan area indicating the importance of graph literacy (GL), we conducted a fully powered, innovative, national, and web-based randomized controlled trial with 203 RNs.

**Objective:**

This study aimed to compare care planning time (CPT) and the adoption of evidence-based CDS recommendations by RNs randomly assigned to 1 of 4 CDS format groups: text only (TO), text+table (TT), text+graph (TG), and tailored (based on the RN’s GL score). We hypothesized that the tailored CDS group will have faster CPT (primary) and higher adoption rates (secondary) than the 3 nontailored CDS groups.

**Methods:**

Eligible RNs employed in an adult hospital unit within the past 2 years were recruited randomly from 10 State Board of Nursing lists representing the 5 regions of the United States (Northeast, Southeast, Midwest, Southwest, and West) to participate in a randomized controlled trial. RNs were randomly assigned to 1 of 4 CDS format groups—TO, TT, TG, and tailored (based on the RN’s GL score)—and interacted with the intervention on their PCs. Regression analysis was performed to estimate the effect of tailoring and the association between CPT and RN characteristics.

**Results:**

The differences between the tailored (n=46) and nontailored (TO, n=55; TT, n=54; and TG, n=48) CDS groups were not significant for either the CPT or the CDS adoption rate. RNs with low GL had longer CPT interacting with the TG CDS format than the TO CDS format (*P*=.01). The CPT in the TG CDS format was associated with age (*P*=.02), GL (*P*=.02), and comfort with EHRs (*P*=.047). Comfort with EHRs was also associated with CPT in the TT CDS format (*P*<.001).

**Conclusions:**

Although tailoring based on GL did not improve CPT or adoption, the study reinforced previous pilot findings that low GL is associated with longer CPT when graphs were included in care planning CDS. Higher GL, younger age, and comfort with EHRs were associated with shorter CPT. These findings are robust based on our new innovative testing strategy in which a diverse national sample of RN participants (randomly derived from 10 State Board of Nursing lists) interacted on the web with the intervention on their PCs. Future studies applying our innovative methodology are recommended to cost-effectively enhance the understanding of how the RN’s GL, combined with additional factors, can inform the development of efficient CDS for care planning and other EHR components before use in practice.

## Introduction

With the proliferation of health care data, an overwhelming amount of information could be made available as robust clinical decision support (CDS) for clinicians in a genuinely helpful format. Despite the length of time that electronic health records (EHRs) have been around, there continue to be reports of unintended consequences from poor design and inadequate testing [1-5] adversely affecting patients and clinicians. Nurses report major frustrations with long documentation time [6-8] and interacting with complicated EHR user interfaces as they seek to locate, understand, apply, and record pertinent information about patients’ care [1]. These frustrations have been linked to nurse burnout and moral distress manifested as work disengagement, dissatisfaction, and no sense of accomplishment [5-8]. Ensuring that CDS are in formats that can decrease documentation time and enhance the quality of decisions could reduce nurse burnout and moral distress. Do-no-harm approaches are needed for identifying and addressing flaws in CDS that can cause negative patient outcomes if undetected before use in practice [9-16]. Building on the findings of our laboratory-based, National Institute of Nursing Research–funded pilot study [17], the primary aim of this web-based, national randomized controlled trial (RCT) was to tailor the CDS format to the graph literacy (GL) of registered nurses (RNs) and examine the impact on nurse care planning efficiency and the adoption of CDS recommendations. Care planning is a vital component of EHRs and a requirement of hospital accrediting bodies (eg, Joint Commission [18]) and for care reimbursement coverage (eg, Centers for Medicare and Medicaid *§482.23(b)(4)* [19]).

A systematic review of 28 studies targeting hospital nurses’ decision-making underscored the promise of CDS, including improved care processes in 7 out of 7 studies, improved usability in 4 out of 4 studies, and improved patient outcomes in 3 out of 4 studies [20]. However, only 2 studies attempted to investigate the CDS format design. One study used a focus group [21] to explore alert types, and the other, an observational study, compared the effect of graphically displayed patient intensive care unit data with those in a tabular format [22]. In the latter, intensive care unit nurses detected more abnormal variables with data presented in the graph format than in the tabular display (*F*_1,119_=13.0; *P*<.05). No significant differences in nurses’ perceived workload were found. In another systematic review of visualizations of patient-reported outcomes, clinicians’ preferences for line graphs, heat maps, or bar charts and objective accuracy of interpretations were mixed [23].

In a study of visual data displays in 2 homecare facilities, nurses understood graphs most easily, followed by tables, line graphs, and spider graphs. Furthermore, nurses with low numeracy and GL had poorer comprehension of the information displayed across all formats. High GL appeared to enhance the comprehension of data regardless of numeracy [24]. In a sample of nonclinicians, the comprehension of statistical information was optimized when participants with high GL were presented with graphs instead of numbers and vice versa [25]. Other studies found that factors influencing participants’ comprehension of health [26] and medical data [27-29] when matched with the format can improve decision accuracy and decision-making time [30,31]. Furthermore, we found no studies other than ours [17,32] that examined the impact of the CDS display format on the time spent in care planning, an important requirement of EHRs in hospital settings by accrediting and funding entities [18,19].

In our prior laboratory-based preclinical simulation RCT, also funded by the National Institute of Nursing Research, we compared the care planning time (CPT) and rates of selecting evidence-based best practice nursing diagnoses, interventions, and outcomes for RNs. The 60 RNs were randomly assigned to 1 of 4 different CDS format conditions: text only (TO), text+table (TT), text+graph (TG), or no CDS [17]. We found significantly higher adoption rates of best practices for all 3 CDS formats compared with the group with no CDS. We also found that the GL of RNs and format type (low GL and TO, medium GL and TT, and high GL and TG) were associated with low CPT. These exploratory findings provided preliminary evidence that RNs with different levels of GL process the care planning CDS formats with varying levels of efficiency indicating the necessity of a confirmatory study [17,32].

**Figure 1:**
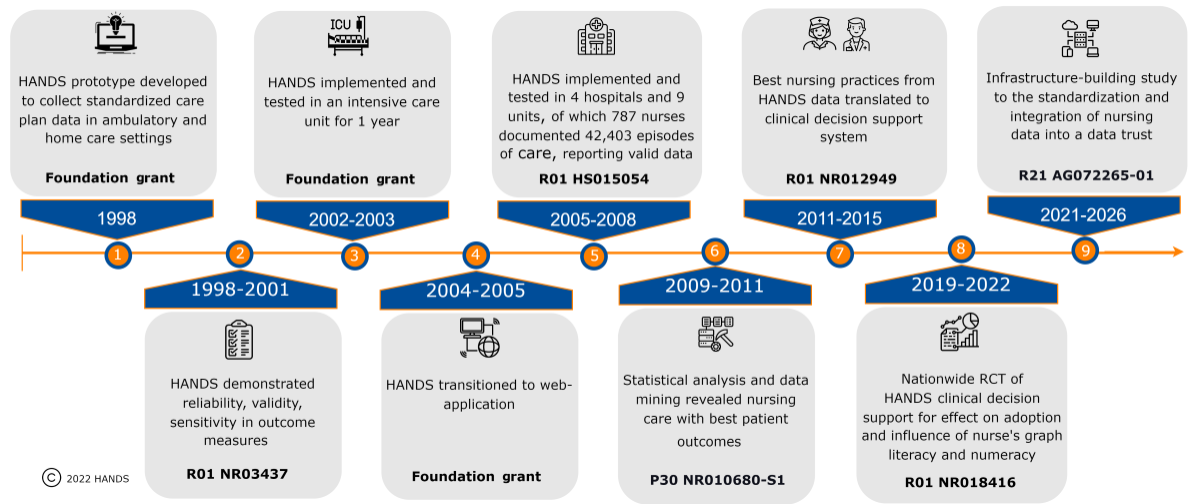
History of HANDS (Hands-on Automated Nursing Data System) research [33].

The primary aim of our nationally representative, web-based simulation RCT reported here was to examine the effects of tailoring (ie, assigning RNs to a format type based on GL) by comparing 203 RNs randomly assigned with CDS format groups (TO, TT, TG, and tailored) on CPT and the adoption of evidence-based best practices. We hypothesized that the tailored CDS group would have faster CPT (primary) and higher CDS recommendation adoption rates. This study is part of our team’s larger research agenda focused on generating standardized nursing data gathered in EHRs, automatically analyzing these data, and returning relevant evidence to the point of care in a CDS format supporting efficient and effective RN decisions. This study is part of a research agenda that began in 1998 with the creation of a research-based nurse care planning system. A synopsis of the team’s work to date is shown in Figure 1 [33].

## Methods

### Design

**Figure 2:**
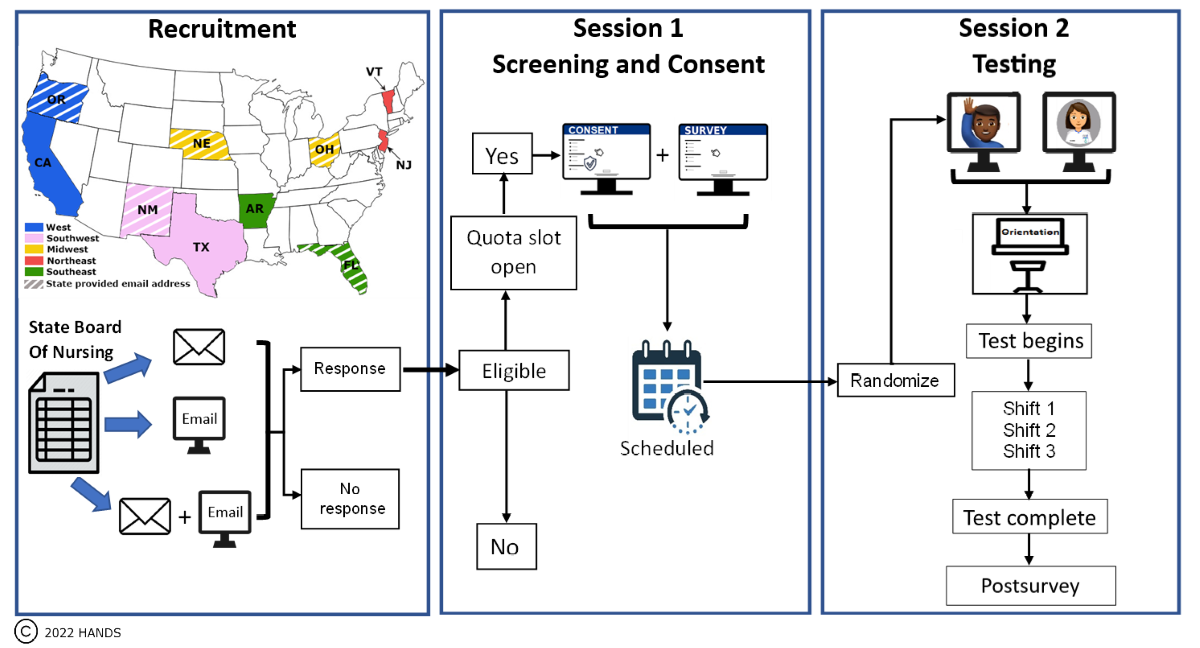
Flow of national web-based study. AR: Arkansas; CA: California; FL: Florida; HANDS: Hands-on Automated Nursing Data System; NE: Nebraska; NJ: New Jersey; NM: New Mexico; OH: Ohio; OR: Oregon; TX: Texas; VT: Vermont.

This study was a national, web-based RCT of 203 RN participants randomly assigned to 1 of the 4 CDS format groups: TO, TT, TG, or tailored. RNs assigned to the first 3 groups interacted with the corresponding CDS format condition, whereas RNs assigned to the tailored CDS group interacted with the CDS format condition matched to their GL scores: TO for those with low GL, TT for those with medium GL, and TG for those with high GL. The score cutoffs can be found in the *Study Measures* section. The study involved 3 phases: recruitment, screening, and consent—session 1; testing intervention; and postsurvey—session 2 (Figure 2). For additional details regarding our method, see our study protocol published elsewhere [32].

### Ethics Approval

This study was approved by the institutional review boards (IRBs) of the University of Florida (IRB201902611) and the University of Iowa (201910213).

### Study Framework

Our study was guided by the Cognitive Load Theory (CLT) [34]. The CLT aims to explain problems when excessive cognitive demands decrease a worker’s performance [35]. The theory posits that human processing capacity is limited and that cognitive load is influenced by differences in individual learners [36], supporting the idea that tailoring information display for individual learners can help decrease the cognitive load.

**Figure 3:**
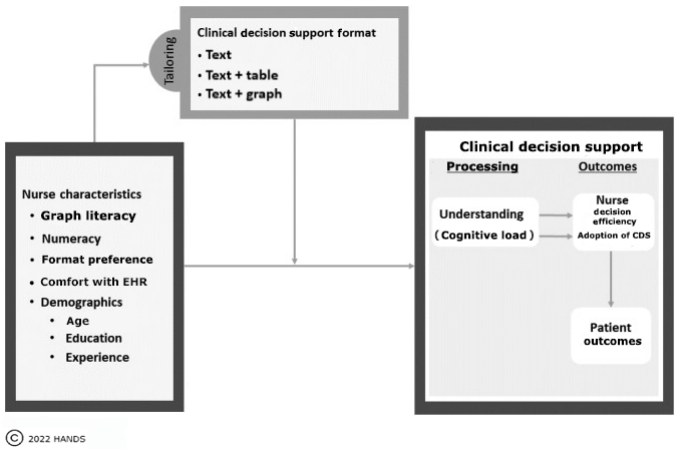
Study conceptual framework guided by the Cognitive Load Theory [28]. CDS: clinical decision support; EHR: electronic health record.

In our study, we recognized that RNs spend much of their day processing large amounts of data (eg, vital signs, pain, laboratory results, and physical assessments) to make clinical decisions. The data processing time combined with multiple competing demands on nurses’ time leads to a high cognitive workload. A suboptimal display of CDS that does not accommodate individual nurses’ learning differences (eg, GL, education, and clinical experience) is expected to increase the cognitive load of RNs and diminish their understanding of the data and the efficiency of decision-making in terms of time (Figure 3).

As depicted in Figure 3, the interaction of the individual RN’s characteristics and the CDS format leads to the cognitive load potentially influencing understanding of the CDS, which in turn affects the decision-making efficiency and adoption of CDS and the patient outcomes. We hypothesized that tailoring the CDS format to the RN’s cognitive characteristics, GL in this study, would reduce the cognitive load and improve the efficiency of care plan decisions.

### CDS Intervention

We devised an innovative approach that enabled us to conduct a cost-effective national study to validate the findings of our pilot study comprising RNs from a large Midwest US metropolitan area [17]. To do this, we converted the laboratory-based version of our CDS intervention into a web-based application that RN participants could securely access and interact with from their PCs, typically at home. Zoom videoconferencing software (Zoom Video Communications) was integrated into the intervention so that the study staff could orient the RN participants and remain present in the background while RNs engaged with the intervention and completed the postsurvey.

The first year of the study was spent reprogramming and iteratively testing the new web-based version of the intervention. Reprogramming was performed by our team programmer and initial testing was conducted by the team. In the final phase of development, the entire procedure for accessing, orienting, and interacting with the intervention was pretested with a sample of 31 RNs (10 from our initial study recruitment cycles and 21 from a convenience sample). This ensured that the intervention operated as intended for a broad range of user setups (internet and computer), enabling a diverse set of RNs to participate from their homes.

**Figure 4:**
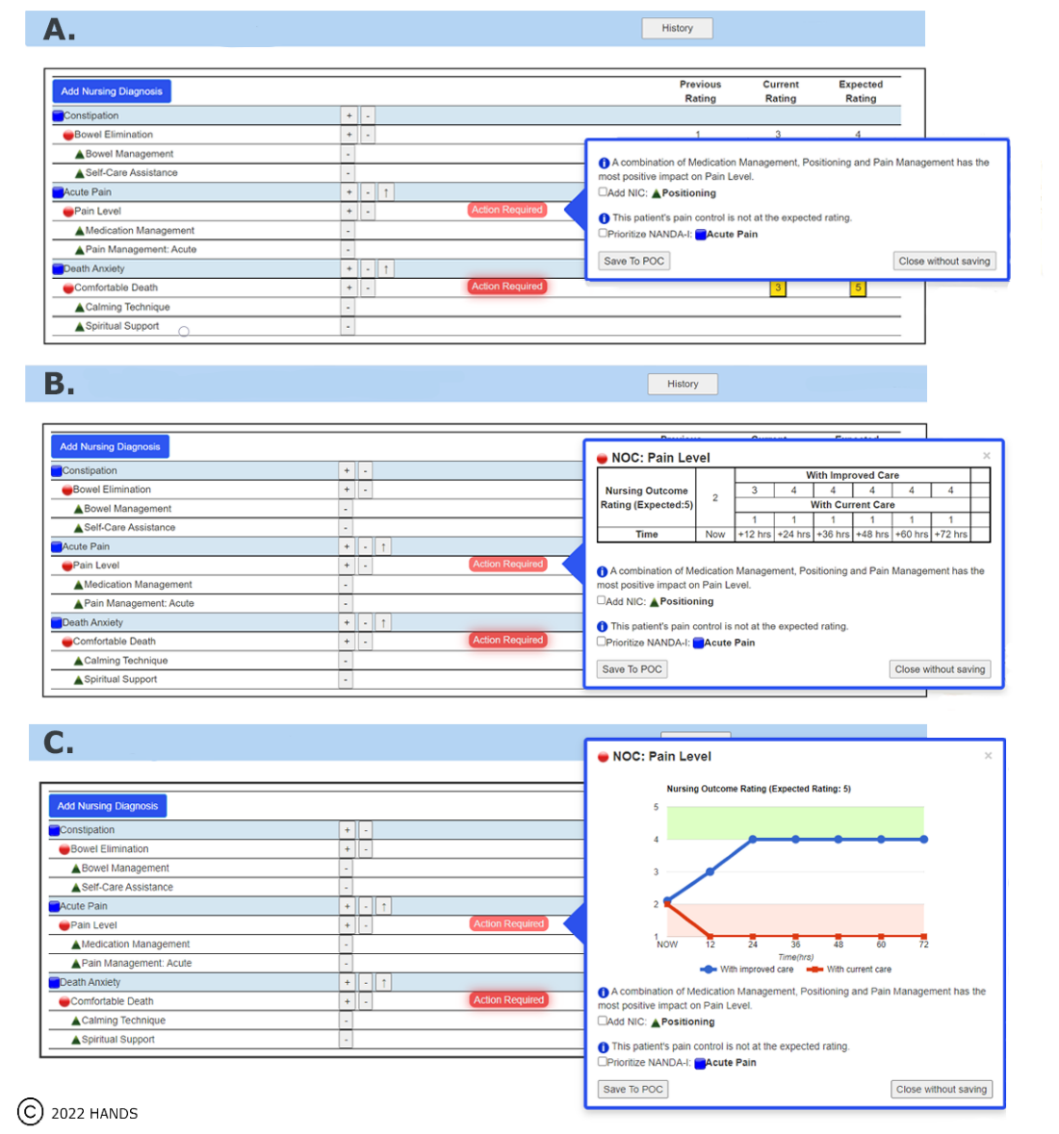
Intervention clinical decision support formats assigned to registered nurse participants: (A) text format, (B) table + text format, and (C) graph + text format. HANDS: Hands-on Automated Nursing Data System; NIC: Nursing Interventions Classification; NOC: Nursing Outcomes Classification; POC: Plan of Care.

The intervention, described in detail elsewhere [32], began with a 10-minute orientation to the study care planning system, an abridged version of the HANDS (Hands-on Automated Nursing Data System) care planning system. The study care planning system used the standardized terminologies of NANDA-I [37], Nursing Outcomes Classification [38], and Nursing Interventions Classification [39] to represent nursing diagnoses, outcomes, and interventions, respectively. Next, the RN was provided with historical information about 2 hypothetical palliative care patients and directed to engage with the care planning system until all care plans were submitted across 3 separate shifts. When a care plan opened on a shift, blinking red buttons would appear indicating a potential need for changes. Once selected, a CDS message displayed in 1 of 3 formats: TO, TT, and TG (Figure 4). All 3 formats contained identical text suggestions coded with NANDA-I, Nursing Outcomes Classification, or Nursing Interventions Classification terms. However, the TT and TG formats also included either a table (TT) or a graph (TG) comparing the projected outcomes of taking or not taking the recommended actions. The RN participants were deliberately not oriented to the care planning CDS and were instead directed to interact with the software as each saw fit. Once all care plans were submitted, the RN completed our postsurvey that immediately followed.

The hypothetical patient scenarios were developed by the investigators (DW and GK) using real patient histories and simulating realistic patient trajectories across time. The CDS recommendations in the study were based on evidence generated by our research team on previous data collected with HANDS under real-time conditions and peer-reviewed literature. An example of Mr Taylor’s history (a hypothetical patient in the study) is shown in Multimedia Appendix 1. There were 9 CDS recommendations generated across 2 patient scenarios during the intervention (Multimedia Appendix 2).

### Recruitment

**Figure 5:**
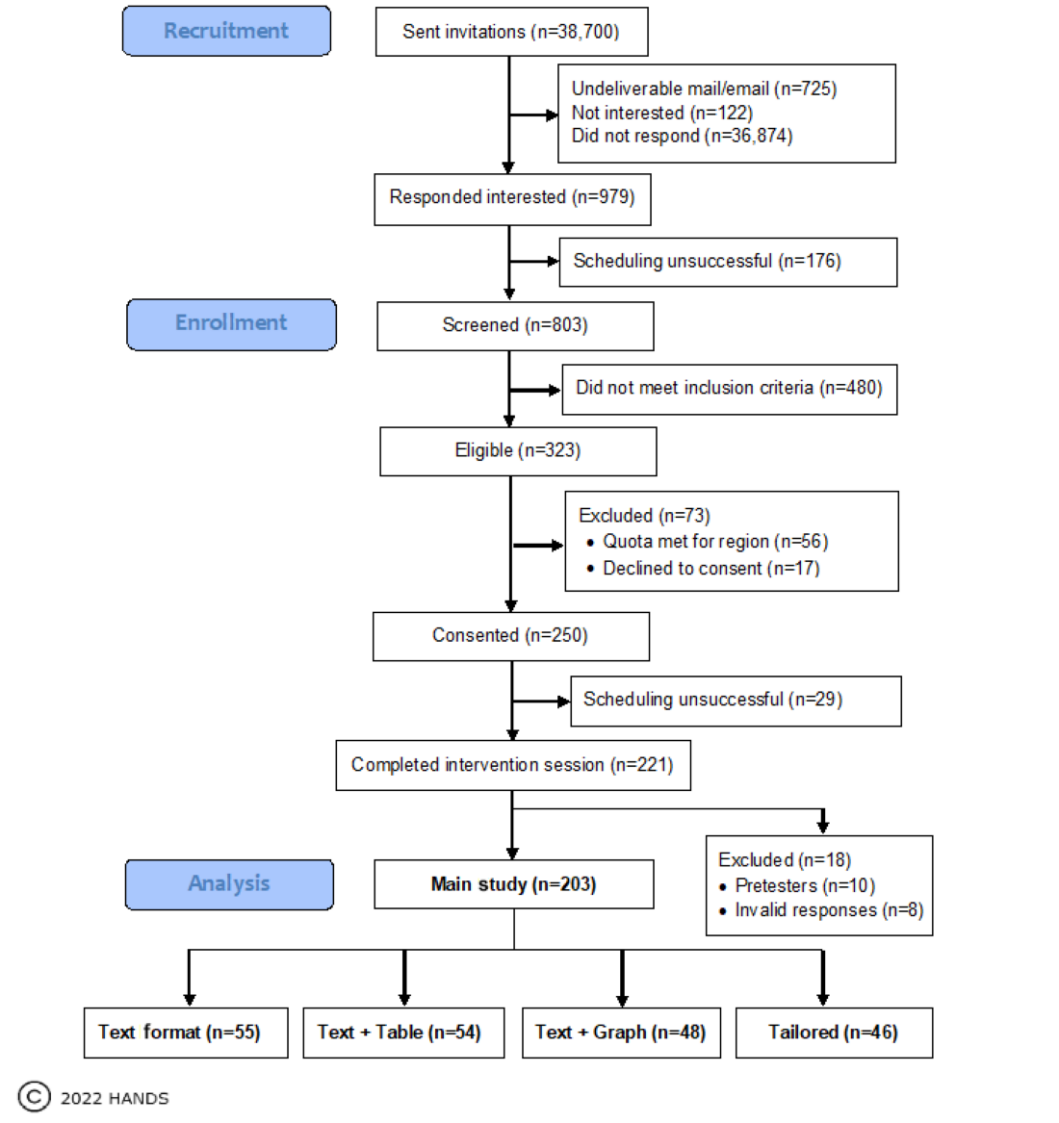
Recruitment CONSORT (Consolidated Standards of Reporting Trials) diagram.

The data reported for this intervention study were collected from RN participants between June 2019 and January 2022 in 2 sessions (Figure 2). To ensure broad representation, RNs were recruited using 10 State Board of Nursing (SBON) publicly available registry lists. The states were selected to represent 5 regions of the country and included some of the most and least populated states and those with high and low population densities: Northeast, New Jersey and Vermont; Southeast, Florida and Arkansas; Midwest, Nebraska and Ohio; Southwest, New Mexico and Texas; and West, California and Oregon. Recruitment occurred across 18 months and included 34 cycles, each using one of three contact approaches: (1) mail only, (2) mixed (mail and email), and (3) email only (Figures 2 and 5).

The 10 SBON lists contained addresses and phone numbers of RNs, with 5 states (Florida, New Mexico, Nebraska, Ohio, and Oregon) also providing email addresses. Potential RN participants from SBON lists without email addresses were contacted through the mail-only approach, and those with email addresses were contacted by either the mixed (mail and email) or email-only approaches. All RNs contacted were randomly selected from these lists and sent recruitment messages with follow-up messaging as per the survey recruitment methods by Dillman et al [40]. The possible messages for the 3 approaches were as follows: 3 for mail only, 5 for mixed, and 4 for email only.

Nurses were eligible if they (1) were aged ≥18 years; (2) possessed an RN license; (3) worked on an adult medical-surgical unit in a hospital setting within the last 2 years; and (4) met technical requirements—access to a tablet, laptop, or desktop computer (<4 years old) with a screen size ≥9” on the diagonal; an internet connection of ≥25 Mbps; had Windows 10 operating system, Apple macOS 10, or newer systems; and had the latest versions of Zoom and Google Chrome or Safari. The exclusion criteria were as follows: (1) did not meet technical requirements and all other inclusion criteria; (2) were unable to complete the study owing to physical or cognitive impairment; or (3) were unable to speak, read, or write English. To further enhance generalizability, quotas were set to ensure the representation of important subgroups: men (goal 20%), racial and ethnic minority group participants (goal 35%), and nurses with Associate’s Degree in Nursing as their highest degree (goal 30%). We set out to recruit approximately 20% from the 5 US regions.

### Sample Size

Our primary aim was to estimate the effect of tailoring on RN CPT. Therefore, we focused on the comparison between the tailored CDS group and each of the 3 nontailored CDS groups: TO, TT, and TG. To achieve a family-wise type-1 error of 5%, we set the cutoff for significance at 0.016 using the Bonferroni adjustment. Using the findings from our pilot study on the association between GL and RN CPT [41], we estimated that tailoring would have an effect size of 0.67. Using the R *pwr* package, we determined that a sample of 200 RNs would provide sufficient (>80%) power to detect the differences between the tailored CDS group and the 3 nontailored CDS groups.

### Randomization

Stratified by GL (low, medium, and high), block randomization was used to assign RNs to 1 of the 4 CDS format groups (TO, TT, TG, or tailored). All 4 groups accessed the same 2 patient scenarios. Randomization was performed by the software automatically. Study staff recruiting and interacting with the RNs and the RN participants themselves were blinded to the group assignment.

### Study Procedures for Sessions 1 and 2

Data collection occurred in 2 sessions. The amount of time needed to complete both sessions was approximately 1 to 2 hours.

1. Session 1: Screening and consent—this session involved screening, consenting (using a web-based process), collecting participant demographics, and assessing the RN participant’s GL score. Data were collected and securely stored in the REDCap (Research Electronic Data Capture; Vanderbilt University) database hosted by the Clinical and Translational Science Institute at the University of Florida, which is Health Insurance Portability and Accountability Act compliant and approved by the IRB of the University of Florida and privacy office for the collection and storage of protected health information. After completing the GL assessment in session 1, eligible RNs in categories under the quota limit were scheduled for session 2 (CDS intervention).
2. Session 2: Experimental CDS intervention—in this session, data related to the experimental intervention and the postsurvey were collected. These data were securely stored in a SQL database server at the University of Florida. No personal information was collected in this session. Before the start of session 2, the project staff verified that each RN participant had access to the required technology, including the Zoom app and an internet browser compatible with the study care planning system. The session began with a standardized video orientation to the user interface and the care planning terminologies, nursing diagnosis [37], nursing outcomes [38], and nursing interventions [39]. To mitigate potential evaluation anxiety of the RN, each participant was reassured that the experiment aimed to test how well the care planning system works and how RNs might respond to it. Its purpose was not to test RNs’ skills or the quality of the care plans. Following the orientation, the RN was provided with a secure URL link via the Zoom chat to access and complete the study intervention and follow-up surveys.

The study intervention occurred across 3 simulated shifts and began with screens showing the current care plans for 2 hypothetical patients. The research assistant verbally delivered the histories of the 2 hypothetical patients. The RN was directed to update the 2 patients’ care plans based on the patient histories and other information that appeared during the intervention. The research assistant observed but did not interact with the RNs as they updated the care plans. After the RN completed the first simulated shift, the intervention included directions for a second and third shift in which the patients’ outcomes and CDS were automatically updated to reflect the RN’s decisions in the prior shift. The RNs viewed the updated outcomes and were asked to apply their clinical judgment when processing the information and prompts displayed (eg, context information, care plan content, and CDS recommendations in the assigned format). Figure 4 provides examples of the 3 CDS formats and Multimedia Appendix 1 provides an example of the historical and handoff information provided during the intervention. After updating the 2 patients’ care plans for all 3 shifts, the RN was directed to complete the 35-item postsurvey. At the end of the study session, the RN was compensated with a US $100 Amazon gift card or 1 credit hour toward a continuing education unit.

### Hypothesis

We hypothesized that the RNs assigned to the tailored CDS group (those presented with the optimal CDS format matched to their GL scores) will have faster CPT and higher CDS suggestion adoption than those assigned a fixed CDS format independent of their GL.

### Study Measures

*Demographics and comfort with EHRs* were collected in session 1—gender (man, woman, or other); race (Alaska Native, American Indian, Asian, Black or African American, Pacific Island, White, or other); Hispanic (yes or no); nursing degree programs completed (diploma, Associate’s Degree in Nursing, Bachelor of Science in Nursing, Master of Science in Nursing entry, Master of Science in Nursing, Doctor of Nursing Practice, or PhD); years of experience; and state of RN license. Comfort with EHRs was assessed using a single item rated on a scale of 1 to 10.

The primary outcome of this RCT was CPT, defined as the total amount of time (in minutes) the RN spent interacting with the care planning software over the 3 simulated shifts. The secondary outcome was the CDS adoption rate, defined as the percentage of CDS recommendations adopted. Each action that the RN took while interacting with the CPS was automatically time-stamped and recorded by our application.

*GL* is a 13-item objective tool with one right answer per item [42]. The tool presents different graph formats (eg, bar and line graphs and pie charts) and asks questions related to interpreting of the information in the graphs. It takes approximately 10 minutes to complete. Reliability and convergent validity were previously assessed and reported (Cronbach α=.85, *r*=0.44). The total score is the number of items answered correctly (range 0-13). In our study, GL was assessed on all RNs before randomization and used to assign tailored CDS group RNs to format conditions as follows: RNs with scores up to 10 were presented with the TO format, those with scores of 11 were presented with the TT format, and the RN with scores ≥12 were presented with the TG format [32]. This tailoring method was motivated by our pilot findings [17].

*Numeracy*, the ease with which individuals can process basic probability and mathematical concepts, was measured objectively with the Numeracy Scale [43]. The Numeracy Scale consists of 11 items (3 general numeracy items and 8 risk numeracy items) that cover probabilities, proportions, and percentages, each having a single right answer. Numeracy was assessed after RNs completed the study intervention.

*Cognitive workload*, the perceived amount of mental processing required to perform clinical tasks in the EHR [44,45], was measured using the NASA Task Load Index instrument (pencil and paper version) after the RNs completed the study intervention. The instrument includes 6 areas (mental demand, temporal demand, physical demand, perception of own performance, effort, and frustration level) related to the responder’s perception of the workload [44,46,47]. It performs well psychometrically (Cronbach α>.80) and has been used extensively to study workload in aviation and other complex industries [46-48], including the health care domain [49-51].

### Statistical Analysis

Session 1 data were stored in REDCap, whereas session 2 data were stored in the SQL databases. Data were imported into the statistical software R (R Foundation for Statistical Computing) for the analysis. Descriptive statistics, including mean, SD, frequency, percentage, and IQR (25th percentile and 75th percentile) were obtained. ANOVA and Fisher exact test were used to examine the variation of variables across groups. Linear regression analysis was used to compare CPT (primary outcome) and CDS adoption rate (secondary outcome) between the tailored CDS group and the 3 nontailored CDS groups and to examine their association with various predictors. For the handling of the small amount (<1%) of missing data, we used the approach of multiple imputations, where multiple completed data sets were generated using the fully conditional specification. Inference was performed on each completed data set and then aggregated using the Rubin rule [52]. CPT was approximately lognormally distributed. A log transformation was applied before the regression analysis of CPT. Statistical significance was set at a *2-sided α of .016* for comparison of the primary outcome between the tailored CDS group and the nontailored CDS groups and .05 for other analyses.

## Results

### Sample

A total of 38,700 RNs were randomly selected from the SBON lists and sent recruitment messages using 1 of the 3 methods: mail only (3159/38,700, 8.16% RNs), mixed (2438/38,700, 6.3% RNs), and email only (33,103/38,700, 85.54% RNs). The response rates were low as expected, with the mixed approach having the highest response rate. Table 1 shows the numbers of RNs contacted by method type, response rates, and completers. Of the 979 RNs who responded positively at least 1 time, 323 (33%) were eligible to participate and 221 (22.57%) RNs (10/221, 4.5% pretesters and 211/221, 95.5% main study participants) completed the full study. Of the 211 RNs (n=56, 26.5% in TO; n=55, 26.1% in TT; n=53, 25.1% in TG; and n=47, 22.3% in tailored) in the main study, 8 (n=1 in TO, n=1 in TT, n=5 in TG, and n=1 in tailored) were excluded from the analysis owing to significant interruption during the session for either real-life circumstances or technical difficulties. Our analysis, therefore, included 203 RNs.

The RN completers were aged 22 to 69 years with a mean age of 40.8 (SD 11.7; median 39) years (34%, 18-34 years; 41%, 35-49 years; 22%, 50-64 years; 3%, >65 years). Of the 203 participants, 20% (n=41) were men, 9.4% (n=19) were Asian, 10.3% (n=21) were Black, 12.8% (n=26) were Hispanic, 74.9% (n=152) were White (n=133, 66% non-Hispanic White), and 5.4% (n=11) were other races. Overall, 73.9% (150/203) of the participants had a Bachelor of Science in Nursing. On average, the participants had 12.3 (SD 10.5) years of RN experience, with 55% (112/203) having up to 10 years, 27% (55/203) having 11 to 20 years, 9% (19/203) having 21 to 30 years, 7% (15/203) having 31 to 40 years, and 1% (2/203) having >40 years of experience, and identified themselves as very comfortable (8.6, SD 1.5 out of 10) working with the EHR. RNs in our study had relatively high GL and numeracy scores, with a mean of 11.0 (SD 1.6) and 8.9 (SD 1.8), respectively. The 2 scores were weakly correlated (*r*=0.26; *P*<.001). The test reliabilities for these 2 scales were 0.70 and 0.82, respectively. Table 2 provides descriptive statistics of the entire sample by format group.

**Table 1:** Recruitment numbers from State Board of Nursing lists by contact method type.

	Mail (n=3159)	Mixed (n=2438)	Email (n=33,103)
Sent, n	3159	2438	33,103
Responded positively, n (%)	245 (7.76)	283 (11.61)	451 (1.36)
Screened, n (%)	206 (6.52)	234 (9.6)	363 (1.1)
Eligible, n (%)	81 (2.56)	104 (4.27)	138 (0.42)
Consented, n (%)	65 (2.06)	84 (3.45)	101 (0.31)
Completed study, n (%)	58 (1.84)	75 (3.08)	88 (0.27)
Pretesters (not part of main study), n	2	8	0
Main study RNs^a^, n	56	67	88

^a^RN: registered nurse.

**Table 2:** Demographics of registered nurse participants (overall and by group).

Characteristics			Overall (n=203)		Tailored (n=46)		TG^a^ (n=48)		TT^b^ (n=54)		TO^c^ (n=55)		*P* value	
Age (years), mean (SD)			40.8 (11.7)		41.4 (10.1)		40.1 (13.1)		41.5 (11.5)		40.2 (12.2)		.90	
Gender (woman), n (%)			162 (79.8)		38 (83)		40 (83)		38 (70)		46 (84)		.29	
**Race, n (%)**														.04
	Asian	19 (9.4)		4 (9)		10 (21)		3 (6)		2 (4)				
	Black	21 (10.3)		7 (15)		4 (8)		3 (6)		7 (13)				
	White	152 (74.9)		31 (67)		30 (62)		47 (87)		44 (80)				
	Other	11 (5.4)		4 (9)		4 (8)		1 (2)		2 (4)				
Ethnicity (Hispanic), n (%)			26 (12.8)		3 (7)		5 (10)		9 (17)		9 (16)		.35	
Education (BSN^d^ or higher), n (%)			150 (73.9)		33 (72)		37 (77)		39 (72)		41 (75)		.92	
Experience (years), mean (SD)			12.3 (10.5)		12.8 (8.6)		12.3 (10.8)		12.9 (11.1)		11.5 (11.2)		.89	
Comfort with EHR^e^, mean (SD)			8.6 (1.5)		8.7 (1.6)		8.4 (1.5)		8.6 (1.3)		8.6 (1.6)		.81	
Graph literacy, mean (SD)			11.0 (1.6)		10.7 (1.9)		11.2 (1.3)		10.9 (1.7)		11.1 (1.4)		.45	
Numeracy, mean (SD)			8.9 (1.8)		9.0 (1.9)		8.7 (2.0)		8.9 (1.8)		8.9 (1.8)		.92	
**Region, n (%)**														.93
	Midwest	38 (18.7)		8 (17)		11 (23)		9 (17)		10 (18)				
	Northeast	34 (16.7)		7 (15)		9 (19)		10 (19)		8 (15)				
	Southeast	40 (19.7)		10 (22)		12 (25)		7 (13)		11 (20)				
	Southwest	45 (22.2)		9 (20)		9 (19)		14 (26)		13 (24)				
	West	46 (22.7)		12 (26)		7 (15)		14 (26)		13 (24)				

^a^TG: text+graph.

^b^TT: text+table.

^c^TO: text only.

^d^BSN: Bachelor of Science in Nursing.

^e^EHR: electronic health record.

### Descriptive Statistics

The descriptive statistics for the 2 study outcomes, CPT and CDS adoption rate, are presented in Table 3. The average CPT was 20.6 (SD 12.0) minutes. On average, the RN participants adopted 89% (SD 15%) of the CDS items, with >81% adopting ≥80% items.

**Table 3:** Descriptive statistics of registered nurse care planning time (CPT) and clinical decision support (CDS) adoption rates (overall and by group).

Outcomes		Overall	Tailored	TG^a^	TT^b^	TO^c^
**CPT (minutes)**						
	Values, mean (SD)	20.6 (12.0)	21.9 (10.5)	22.9 (11.6)	20.5 (16.3)	17.8 (7.4)
	Value, IQR	13.1-25.1	14.1-28.0	14.9-27.3	12.0-22.9	12.1-22.5
	Values, range	5.7-123.4	9.2-63.7	7.6-62.4	5.7-123.4	7.0-37.3
**CDS adoption rate**						
	Values, mean (SD)	0.89 (0.15)	0.87 (0.17)	0.88 (0.13)	0.90 (0.16)	0.90 (0.13)
	Value, IQR	0.82-1	0.80-1	0.82-0.99	0.88-1	0.85-1
	Values, range	0.22-1	0.33-1	0.45-1	0.22-1	0.43-1

^a^TG: text+graph.

^b^TT: text+table.

^c^TO: text only.

### Statistical Analyses

Outputs of the regression analysis comparing the 3 groups assigned a fixed CDS format with the tailored CDS group are presented in Table 4. Our hypothesis was that relative to the tailored group, the other 3 groups would have higher CPT and lower adoption rates. The differences between the nontailored CDS groups and the tailored CDS group were not statistically significant below the cutoff (2-sided Cronbach α at *.016* for the primary outcome and .05 for the secondary outcome) in this sample for either the CPT or the CDS adoption rate. Regression analysis, including RN characteristics (age, gender, race, etc) as covariates yielded the same conclusion.

**Table 4:** Regression analyses of registered nurse care planning time (CPT) and clinical decision support (CDS) adoption rates comparing fixed format groups with tailored group.

Outcome and group (reference=tailored)		Estimate	SE	*t* test (*df*)	*P* value
**CPT^a^**					
	TG^b^	0.027	0.098	0.274 (197)	.78
	TT^c^	−0.126	0.095	−1.324 (196)	.19
	TO^d^	−0.194	0.094	−2.056 (197)	.04
**CDS adoption rate^e^**					
	TG	0.003	0.031	0.107 (197)	.92
	TT	0.027	0.030	0.903 (197)	.37
	TO	0.024	0.030	0.825 (197)	.41

^a^*R*^2^=0.03.

^b^TG: text+graph.

^c^TT: text+table.

^d^TO: text only.

^e^*R*^2^=0.01.

To gain further insight into the impact of tailoring, we examined the premise that the TO format was optimal for participants with low GL, TT format was optimal for those with medium GL, and TG format was optimal for those with high GL. For this exploratory analysis, we focused on CPT, which was the primary outcome of our study. We conducted regression analyses to determine the effects of CDS format on CPT of RNs in each GL category (low, medium, and high). Each GL category had 3 groups, those who interacted with the format matched to their GL and those who interacted with one of the other 2 formats, for example, the 3 low GL groups included those who interacted with the TO (matched format) and those who interacted with TT or TG format. An RN could be exposed to the matched GL format by being assigned to the tailored CDS group or to one of the other 2 formats through random assignment. We hypothesized that RNs assigned to the matched format would have a lower CPT. For RNs with low GL, CPT was higher for those given CDS in the TG formats than in the matched format (*P*=.01). Other comparisons of CPT under the presumed optimal (GL-matched) format and alternative formats did not show significant differences (Table 5).

**Table 5:** Regression analyses of the effects of clinical decision support (CDS) format on registered nurse’s care planning time in each graph literacy (GL) category.

GL category and format		Estimate	SE	*t* test (*df*)	*P* value
**Low (n=57)^a^**					
	TG^b^ vs matched	0.413	0.155	2.668 (52)	.01
	TT^c^ vs matched	−0.044	0.146	−0.299 (51)	.77
**Medium (n=67)^d^**					
	TG vs matched	−0.133	0.149	−0.894 (62)	.37
	TO^e^ vs matched	−0.132	0.143	−0.920 (62)	.36
**High (n=79)^f^**					
	TT vs matched	−0.152	0.128	−1.183 (74)	.24
	TO vs matched	−0.181	0.125	−1.449 (74)	.15

^a^*R*^2^=0.13.

^b^TG: text+graph.

^c^TT: text+table.

^d^*R*^2^=0.02.

^e^TO: text only.

^f^*R*^2^=0.03.

We also examined the variables that could be associated with CPT under various CDS formats using regression analysis (Table 6). A higher comfort level with EHR was associated with lower CPT in the TT and TG formats. In the TG format, higher GL and younger age were also associated with shorter CPT.

Finally, we compared the perceived cognitive workload of using the CDS system reported by the participants in the 4 CDS format groups. The mean and SD by group for each item and the overall workload are presented in Table 7. The test reliability of this scale was 0.85. The difference across groups was not statistically significant on workload items or overall score in this sample (all *P*>.05).

**Table 6:** Regressions examining the potential associations of other variables to care planning time by the clinical decision support format.

Format and predictor			Estimate		SE		*t* test (*df*)		*P* value
**Text only (n=69)^a^**									
	Age (years)	−0.009		0.005		−1.814 (60)		.08	
	Gender (man vs woman)	−0.072		0.152		−0.473 (60)		.64	
	Education (BSN^b^ vs ADN^c^)	−0.081		0.127		−0.634 (60)		.53	
	Graph literacy	−0.016		0.032		−0.513 (60)		.61	
	Numeracy	−0.035		0.033		−1.039 (60)		.30	
	Comfort with EHR^d^	−0.041		0.035		−1.163 (60)		.25	
**Text+table (n=70)^e^**									
	Age (years)	−0.006		0.005		−1.175 (61)		.24	
	Gender (man vs woman)	−0.123		0.133		−0.931 (61)		.36	
	Education (BSN vs ADN)	0.041		0.122		0.339 (60)		.74	
	Graph literacy	−0.015		0.038		−0.402 (61)		.69	
	Numeracy	0.010		0.029		0.335 (60)		.74	
	Comfort with EHR	−0.180		0.041		−4.360 (61)		<.001	
**Text+graph (n=64)^f^**									
	Age (years)	0.012		0.005		2.454 (55)		.02	
	Gender (man vs woman)	0.237		0.148		1.604 (55)		.12	
	Education (BSN vs ADN)	−0.080		0.138		−0.579 (55)		.57	
	Graph literacy	−0.102		0.044		−2.315 (55)		.02	
	Numeracy	0.000		0.032		0.006 (55)		.99	
	Comfort with EHR	−0.074		0.037		−2.030 (55)		.047	

^a^*R*^2^=0.12.

^b^BSN: Bachelor of Science in Nursing.

^c^ADN: Associate’s Degree in Nursing.

^d^EHR: electronic health record.

^e^*R*^2^=0.25.

^f^*R*^2^=0.28.

**Table 7:** Workload comparisons by the clinical decision support group.

Variable	Tailored	TG^a^	TT^b^	TO^c^	*P* value
Mental demand, mean (SD)	42.7 (23.1)	47.1 (27.2)	40.8 (22.3)	45.6 (23.7)	.55
Physical demand, mean (SD)	15.5 (19.7)	19.6 (24.0)	15.2 (15.3)	14.3 (15.8)	.51
Pace of task, mean (SD)	34.4 (24.4)	36.1 (28.7)	28.9 (24.3)	31.9 (25.3)	.52
Success of task, mean (SD)	72.5 (18.9)	66.0 (24.6)	68.7 (23.2)	66.9 (18.0)	.46
Effort needed, mean (SD)	39.2 (22.7)	45.8 (26.1)	38.7 (23.9)	44.9 (23.4)	.30
Stress or irritation, mean (SD)	24.5 (21.8)	27.6 (27.9)	31.9 (27.0)	35.9 (28.3)	.15
Overall workload, mean (SD)	38.1 (12.7)	40.4 (17.0)	37.4 (13.3)	39.9 (13.1)	.66

^a^TG: text+graph.

^b^TT: text+table.

^c^TO: text only.

## Discussion

### Overview

In the current national RCT with 203 hospital RNs, we found that our hypotheses were not supported. We expected the tailored CDS group to outperform the nontailored CDS groups on time spent on care planning and adoption of CDS evidence-based recommendations, but this did not occur. Our exploratory analysis found that comfort working with EHR was associated with less time spent on care planning. Older age and lower GL were associated with longer decision time when evidence was presented in a TG format, whereas such associations did not hold when evidence was presented in a TO or TT format. There were also no significant differences found across groups when comparing the RNs’ perceived cognitive workload of using the assigned CDS. Perhaps the most important outcome, however, is that this study demonstrated the feasibility of an innovative approach for conducting a national RCT on a prototype EHR component via the internet. This success provides a viable approach for pretesting EHR components before integration into practice with truly diverse representative samples of potential users under simulated conditions, thereby avoiding unintended risks to patients.

### Principal Findings

#### Overview

Currently, >96% of general acute care hospitals use EHRs [53] with nurses spending an increasing proportion of their time documenting their care. Hospital EHRs with poorer usability have significantly higher odds of nurse burnout, intention to leave, inpatient mortality, and 30-day readmission than those with better usability [54]. It is therefore critically important to identify novel technical solutions that support a reduction in documentation time that can also enhance and not compromise the quality of care. Our study addressed these needs by testing the impact of tailoring CDS (added to the EHR care planning component) in which RNs were assigned different CDS formats based on their GL with an adequately powered sample.

#### CPT Findings

As noted, we did not find significant differences in CPT between the tailored group (assigned by GL: low=TO, medium=TT, and high=TG) and those assigned randomly to these 3 CDS formats. When examining CPT for RNs by GL, however, we found that RNs with low GL had significantly higher CPT when interacting in the TG format versus when interacting with the TO format (hypothetical optimal format for RNs with low GL). There were no significant differences found for RNs with medium or high GL between hypothetical optimal formats and other formats. These findings provide insight into why the tailored CDS group did not perform better. However, for those with a medium or high GL, other factors may be more important when tailoring CDS formats. In our previous study, we found that higher subjective numeracy and GL were associated with less time spent on care planning for the TT and TG conditions, respectively [17]. This study replicated our previous finding that a higher GL was associated with shorter CPT in TG condition. The robustness of the association between GL and time needed to process TG CDS information suggests the importance of considering the RN’s GL when considering whether to present evidence in a graph format. Other researchers have found that a higher GL is associated with greater comprehension of the data [24].

We also know from our team’s earlier research that GL varies among RNs [17,55]. Other researchers have also found that incorporating graphic design in decision aids decreased the information processing time [30,31,56,57]. Although they did not address time, Dowding et al [24] found that nurses with low numeracy and GL had poorer comprehension of the visualized data. Although our findings on CPT did not support our hypotheses, our analyses indicate the need for considering the RN’s GL and other characteristics when designing CDS that can reduce documentation time. Specifically, there is a need to enhance our knowledge of the combination of features (eg, type of data, user characteristics such as GL, and display format) to apply when constructing CDS conditions that will lead to decreased decision and documentation time. For example, it may be important to provide plain text CDS information that is intuitively clear for RNs with low GL and to create educational interventions that increase the RN’s GL and ability to quickly process visual displays of numerical data as presented in graphs.

#### Adoption of CDS Suggestions

Another central finding was that, on average, participants adopted the vast majority of the CDS recommendations offered. This is consistent with previous evidence suggesting that clinicians are willing to adopt evidence-based recommendations suggested to them via CDS [17]. We hypothesized that adoption of CDS recommendations would be higher among the tailored CDS format group but found no significant difference in these rates across the 4 groups. Given that the adoption of recommendations was approximately 90% across all groups, there may be a ceiling effect on adoption rates, limiting variation across groups. It is important to note that all 3 CDS format types, TO, TT, and TG, included identical text recommendations with a table or graph also displaying the TT and TG formats projecting the patient’s outcomes (eg, if CDS recommendations were adopted or not adopted). The CDS recommendations, for the most part, were simple and involved a small number of variables (Multimedia Appendix 2), which could have contributed to the uniformly high level of CDS adoption across formats. In addition, we only tested a simple line graph in our study. Others have found that inclusion of easy-to-comprehend visualizations within CDS can enhance understanding and confidence in the recommendations and improve adoption [58-60].

If adoption rates are similarly high when evidence-based CDS is implemented in clinical practice, one would expect patient care outcomes to be improved [17]. Future research in clinical settings measuring real patient outcomes is needed to validate optimization with study designs that support causal inferences. Research on the prevalence of inappropriate or high risk (eg, early mobility for patients on extracorporeal membrane oxygenation) CDS recommendations and the associated unintended consequences if selected is also needed. Several important questions should be considered: What combination of CDS format, data type, and RN characteristics (including GL) support the recognition of flawed recommendations? and What mechanisms are needed to ensure an effective balance between RNs assuming all CDS suggestions are correct versus RNs being overly vigilant (adding to decision-making time) in scrutinizing CDS that may be not erroneous?

#### Innovative Methodology

A key innovation of our research was conducting a national RCT remotely using internet-based communication tools (eg, Zoom). This allowed us to securely test the intervention with a diverse, representative sample of RNs in their homes on their PCs. Although we are not the first to use internet-based tools in an RCT [61-64], we are the first to recruit RNs to test CDS remotely. Our innovative testing method demonstrated the feasibility of refining technical innovations such as the RN care planning CDS examined in this study before implementation in practice. As we found with this study, the CDS is not yet ready to use in practice. Our method would also be suitable for preclinical testing of other EHR components and upgrades, for which standardization and interoperability are desired. To date, the methodologies for building many technical health care solutions (eg, EHR components and upgrades) have given insufficient attention to full testing and establishing universal acceptability with users before implementation into practice [65]. As a result, clinicians are continually challenged with integrating technical additions to EHRs into their workflow that may bring little value, require additional time for use, and cause unintended errors and consequences [5,31,66,67]. Furthermore, fixing poorly designed technical add-ons once implemented into practice is difficult and expensive. In addition to those related to fixing the technical design issue, other sunk costs include reductions to efficiency, lowered quality of care, and clinician turnover related to burnout [5].

As generalizability was a priority for establishing the potential broad applicability of our findings, our team translated the pilot laboratory version of the intervention into a web version that could be tested using a national sample of RNs from their homes. The intervention was successfully translated and delivered consistently, ensuring fidelity of the intervention throughout the study. Our recruitment strategy attracted a balanced and diverse national sample of RNs with recent experience in adult hospital units. The RNs contacted for potential participation were randomly selected from 10 state board lists, representing 5 regions of the country. Furthermore, we intentionally oversampled important minority groups (men and Asian, Black or African American, and Hispanic RNs) within the profession that are expected to expand in the years to come. As a result, our sample had a higher proportion of these groups than those found in the 2020 Nursing Workforce Survey [68] among all licensed RNs.

### Past Research and Future Directions

As noted earlier, reports of unintended consequences from poor design and inadequate testing [1-5] of EHRs continue to adversely affect patients and clinicians. Nurses report major frustrations with long documentation time [6-8] and interacting with complicated EHRs to locate and use pertinent information for high-quality decisions [1]. These frustrations are linked to RN work disengagement, dissatisfaction, and no sense of accomplishment [5,7,8]. The care planning component of EHRs is crucial for helping the patient’s health care team coordinate care across time and is also a requirement for accreditation and funding [18,19]. Reducing the care plan documentation time while increasing its quality remains a high priority goal of our research. The current national RCT was designed to build on our pilot findings that found a significant reduction in CPT (9%-20% documentation time) and significantly higher adoption rates of best practices for the CDS groups than the “no CDS” group. The pilot study was conducted in a laboratory and tested under simulated conditions with 60 RNs from a large Midwest metropolitan area.

Building on our pilot findings, we set out to determine whether tailoring assignment to a CDS format by GL would further improve the documentation time and the potential quality of care plans (eg, adoption rates of evidence-based CDS recommendations) in a national RCT of 203 RNs. We purposefully sought to ensure that our findings would represent the diversity of RNs in the United States employed in hospital medical-surgical units. To do this, we implemented an innovative recruitment method and deployment of our intervention. We used 10 SBON lists to randomly identify RNs eligible for our study (Figure 2). The pilot face-to-face laboratory version of our intervention was converted to a web version that was made accessible to RNs with their PCs via the internet. In this RCT, we showed that conducting such studies virtually was feasible and could greatly lower the cost and time commitment needed for travel, multiple IRB approval, etc, associated with a typical multisite study.

Although our RCT hypotheses were not supported, this study replicated our previous findings that low GL was associated with higher CPT in the TG condition. The robustness of these associations, given the high quality of our sample, suggests the need to consider the GL of RNs when designing and integrating visual displays such as graphs into care planning CDS. Our findings are supported by others who also found an association between higher GL and reduced decision time [30,31,56,57].

In summary, although our hypotheses did not hold, the results of this study offer important knowledge for future directions. Our findings suggest a clear need for further research to establish the combination of factors to consider when using visual displays in the EHRs for specified purposes. Although others have examined clinician format preferences and the relationship of visual display type to the accuracy of some types of data, research has yet to establish the conditions (eg, setting, clinician characteristics, data types, and purpose of use) that collectively affect the achievement of desired outcomes. Although our tailoring scheme did not reduce RN documentation time for care planning, we confirmed the relationship between GL and documentation time in our pilot study. In the future, we will continue using our innovative preclinical recruitment and national testing methods to further isolate the factors [69,70] that decrease the documentation burden while increasing the quality of care planning, a crucial component of the EHRs [18,19]. Our future work will build on findings from the RCT that indicate a need to better understand how the RN’s GL can be used to enhance CDS to achieve our desired outcomes. Another possible direction of future research is to conduct a structural equation modeling analysis of models informed by the CLT to fully explore the complex relationship between RN characteristics, CDS formats, care planning efficiency, and CDS adoption.

### Limitations

Our study has some limitations that should be considered as study findings are interpreted. First, although our rigorous recruitment processes produced a nationally diverse population of RNs and important subgroups within, our overall response rate was low indicating potential selection bias. The age range of our participants was lower than the national statistics. The participating RNs may also be atypical in their interest in research in general and health information technology in particular. However, our participants may well represent the workforce 5 years from now and thus our outcomes once further validated will apply to the workforce of the day. Second, this study was conducted during the COVID-19 pandemic. The very high workload and stress placed on RNs might have had an impact on the response rate and on the representativeness of the participating RNs. Third, testing novel EHR features in a simulation environment provides important insight but is only one step toward improving EHR in a clinical setting. Fourth, RNs might have evaluation anxiety that affected how they interacted with the CDS system even though we took great care in the study to mitigate such anxiety. Fifth, RNs’ comfort with EHR was assessed with a single item and found to impact documentation time. Future studies are needed to validate this finding and to investigate the effect of an RN’s prior experience with CDS, which might be more relevant than comfort with EHR in general. Finally, we focused on matching CDS formats to RN characteristics. In clinical practice, it will also be important to take into account other characteristics that we have highlighted above when choosing data visualization formats.

## Appendix

Multimedia Appendix 1 Example of hypothetical patient information and handoff directions shared with registered nurses (RNs) during the intervention.

Multimedia Appendix 2 Nine clinical decision support recommendations in the study.

Multimedia Appendix 3 CONSORT e-HEALTH checklist (V 1.6.1).

## Abbreviations

CDS: clinical decision support
CLT: Cognitive Load Theory
CPT: care planning time
EHR: electronic health record
GL: graph literacy
HANDS: Hands-on Automated Nursing Data System
IRB: institutional review board
RCT: randomized controlled trial
REDCap: Research Electronic Data Capture
RN: registered nurse
SBON: State Board of Nursing
TG: text+graph
TO: text only
TT: text+table

## Data Availability

The study data were collected in 3 phases and are stored securely in different locations. To simplify access and use for interested researchers, the data will be made available upon request.
