# Supplementary figures and images for: Examining Care Planning Efficiency and Clinical Decision Support Adoption in a System Tailoring to Nurses’ Graph Literacy: National, Web-Based Randomized Controlled Trial
Source: J Med Internet Res. 2023 Aug 11;25:e45043. doi: 10.2196/45043 (PMC10457701; doi:10.2196/45043)

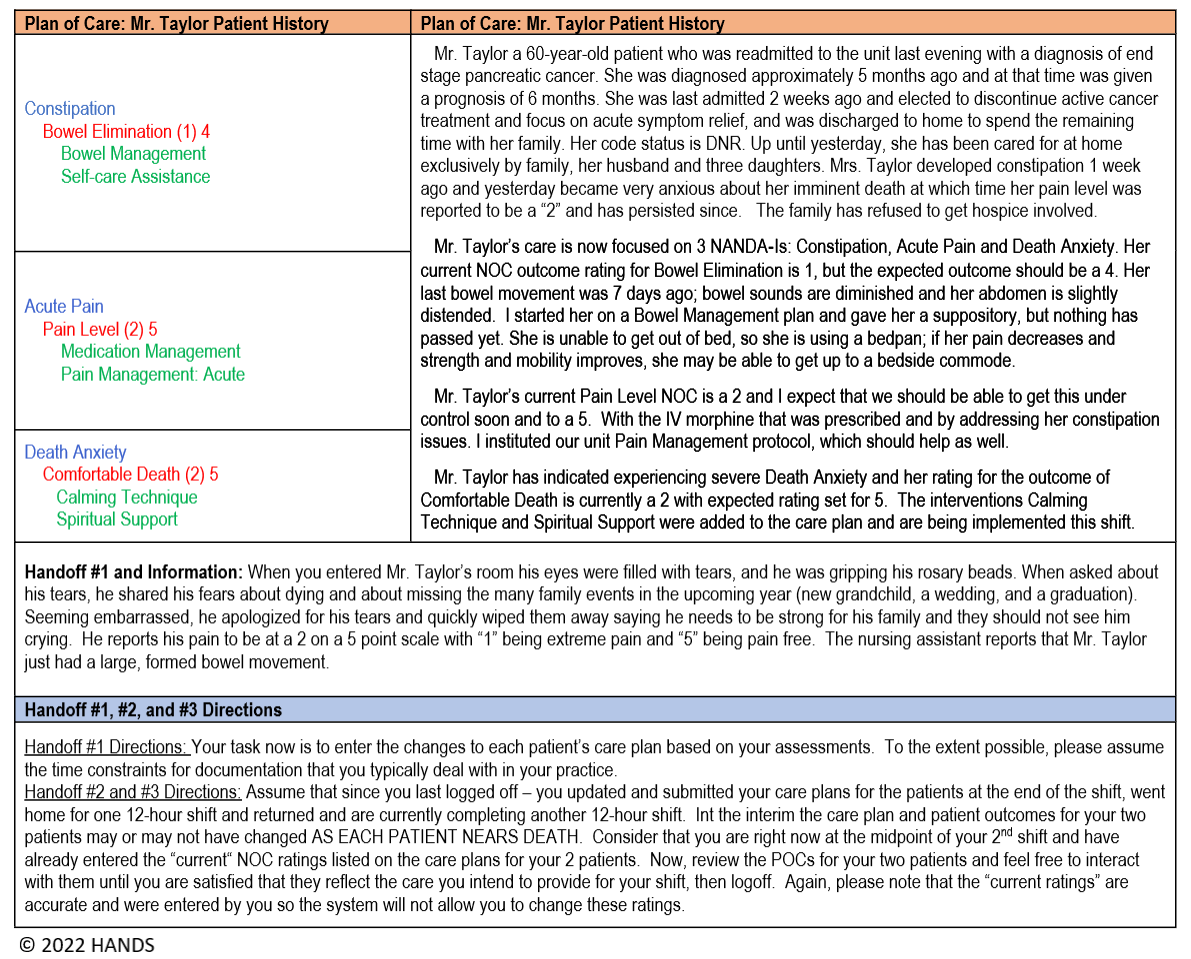

Supplement: Multimedia Appendix 1 [file jmir_v25i1e45043_app1.png]

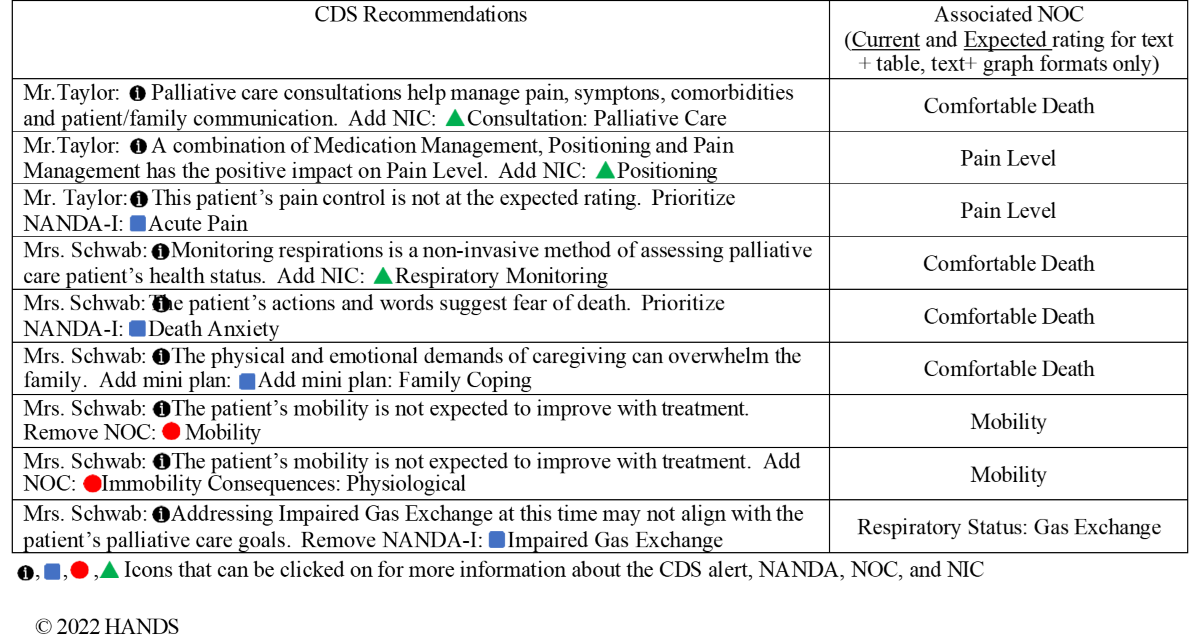

Supplement: Multimedia Appendix 2 [file jmir_v25i1e45043_app2.png]
